# The Rise of Synaptic Density PET Imaging

**DOI:** 10.3390/molecules25102303

**Published:** 2020-05-14

**Authors:** Guillaume Becker, Sylvestre Dammicco, Mohamed Ali Bahri, Eric Salmon

**Affiliations:** GIGA-Cyclotron Research Center-in vivo imaging, University of Liège, Allée du 6 Août, B30, 4000 Liege, Belgium; sdammicco@uliege.be (S.D.); m.bahri@uliege.be (M.A.B.); eric.salmon@uliege.be (E.S.)

**Keywords:** SV2A protein, PET radiotracers, synaptic loss, radiochemistry, preclinical development, clinical outcomes

## Abstract

Many neurological disorders are related to synaptic loss or pathologies. Before the boom of positrons emission tomography (PET) imaging of synapses, synaptic quantification could only be achieved in vitro on brain samples after autopsy or surgical resections. Until the mid-2010s, electron microscopy and immunohistochemical labelling of synaptic proteins were the gold-standard methods for such analyses. Over the last decade, several PET radiotracers for the synaptic vesicle 2A protein have been developed to achieve in vivo synapses visualization and quantification. Different strategies were used, namely radiolabelling with either ^11^C or ^18^F, preclinical development in rodent and non-human primates, and binding quantification with different kinetic modelling methods. This review provides an overview of these PET tracers and underlines their perspectives and limitations by focusing on radiochemical aspects, as well as preclinical proof-of-concept and the main clinical outcomes described so far.

## 1. Introduction

The synaptic vesicle glycoprotein 2A (SV2A) has been studied for three decades which were punctuated by important milestones. Among others, the discovery by Lynch et al. of SV2A being the binding site of the first-in-class antiepileptic drug levetiracetam is one of these significant steps [1]. The most recent milestone is undoubtedly the current possibility to study SV2A in vivo via PET imaging with specific radiopharmaceuticals. The protein is ubiquitously expressed throughout the brain and is known to be essential for proper brain development and functioning [2,3]. Numerous functions have been hypothesized for SV2A and were recently reviewed [4]. It is noteworthy that pharmacological modulation of SV2A protein may have an impact on Alzheimer’s disease (AD) progression [5,6]. Besides the obvious importance of SV2A in epilepsy, AD is a perfect illustration of the clinical interest of synaptic density quantification [7,8]. Before the onset of synaptic density PET imaging, the quantification of synaptic density in brain tissue was performed using immunohistochemistry of key proteins located in the pre- or postsynaptic neurons, such as synaptophysin, or using electron microscopy. Terry et al. used the immunohistochemistry of synaptophysin and demonstrated a lower synaptic density in the midfrontal and inferior parietal cortices of AD patients. Moreover, they reported a positive correlation between the synaptophysin optical density (as an index of synaptic density) and the mini-mental state examination (MMSE) score of the patients [7]. More than a decade later, Scheff et al. counted the synapses in the hippocampus of AD patients using electron microscopy and found a decreased synaptic density in early AD compare to MCI and healthy controls. Consistently with previously mentioned results, Scheff et al. described a positive correlation between the number of synapses in the hippocampus and the MMSE score [8]. Another pertinent example consists in Parkinson’s disease of which the synaptopathy in mainly consists in dysfunction of dopaminergic nigrostriatal terminals and corticostriatal synapses, probably due to the toxic effect of misfolded α-Synuclein. In that case, the majority of genes implicated in PD have a critical role in presynaptic function and thus presumably lead to synaptic dysfunctions. With this in mind, the underlying concept is that PET imaging of synaptic density could be achieved by targeting proteins embedded in synaptic vesicles thanks to appropriate specific radiotracers. Strongly supported by the experience of UCB Pharma in the development of SV2A ligands, several PET research centres embarked on the development of SV2A PET radiotracers. In this review, we return to this “success story” with an emphasis on updated radiochemical considerations of available PET tracers for SV2A. In a second part, we summarize the preclinical developments that were made with these radiotracers, and finally, we report the major clinical outcomes achieved by the existing PET radiotracers.

## 2. Radiochemistry of SV2A PET Radiotracers

**Figure 1:**
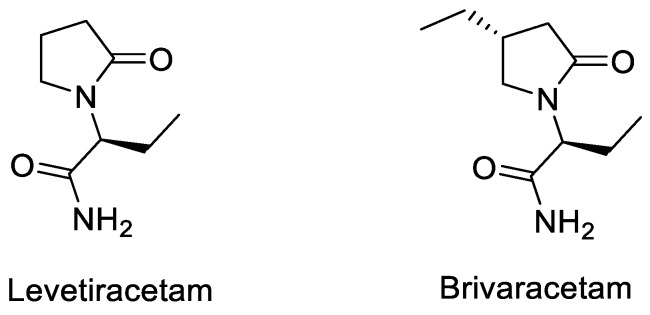
Two antiepileptic drugs: Levetiracetam and Brivaracetam.

Over the last decades UCB has developed several effective antiepileptic drugs sharing the same mode of action for binding SV2A: levetiracetam (Keppra^®^) and brivaracetam (Briviact^®^) (Figure 1) [9,10,11,12].

After further exploration, the acetamide motif was replaced by different heteroaryl groups as imidazole and pyridine derivatives mimicking the acetamide motif [13]. The modification of the substitution of the alky group on the lactam function was also studied and revealed that (poly)-fluorinated aryls provided the best affinities at SV2A obtained so far.

Quantification of SV2A-PET signal aims to provide a measure of brain synaptic density. Specific requirements for designing CNS PET ligands have been recently reviewed extensively [14,15,16,17]. Several parameters are required to obtain a good PET tracer. The radioligand must have a high affinity for the target (nanomolar or subnanomolar) and a *B_max_*/*Kd* ratio in a suitable range to measure a specific signal in vivo (above 10). It has to be able to cross the BBB to reach its target in the CNS. Typically, the LogP of the compound should be between 1 and 3. Since the target density is usually low, the molar activity of the tracer has to be as high as possible. Finally, the radiosynthesis of the tracer with a PET radionuclide as fluorine-18 or carbon-11 must be fast and easily implemented.

Initially, in 2014, the radiochemical synthesis of levetiracetam was performed with carbon-11 (Table 1, entry 1) by Cai et al. but the affinity for SV2A of levetiracetam (Ki = 2.5 µM) was too low for in vivo imaging application [18]. Several other candidates presenting good affinity for SVA were also developed by UCB and were labelled with fluorine-18 and carbon-11: [^18^F]UCB-H, [^11^C]UCB-A and [^11^C]UCB-J (Table 1, entry 2–5). All these compounds were synthesized in partnership with PET centers, in the University of Liège for [^18^F]UCB-H, in Uppsala University in Sweden for [^11^C]UCB-A and finally, in Yale University in New Haven for [^11^C]UCB-J. These compounds have two enantiomeric forms due to the chiral carbon on the lactam ring. In every case, the *R* form presents best affinity for the SV2A target.

The first published radiotracer was [^18^F]UCBH. A multi-step synthesis was described in 2013 by Aerts et al. involving nucleophilic ^18^F-labelling (entry 2) of a pyridine derivative precursor followed by reductive amination, reduction and ring closure reaction [19,20]. This approach afforded UCB-H with a no decay corrected radiochemical yield (ndc RCY) of 30%. The duration of the synthesis including the purification and formulation steps was of 150 min and the molar activity of 96.2 GBq.µmol^−1^. This process was fully automated on a FASTlab synthesizer and was until recently used in the Liège laboratory for cGMP routine production of the radiopharmaceutical.

In 2014, an alternative at this time consuming multi step synthesis was proposed by the same laboratory and greatly simplified the automation process [21]. This late stage approach (entry 3) requires the labelling of a Pyridyl(4-methoxypehnyl)iodonium triflate salt precursor according to the Pike methodology [22]. In order to improve the yield of the labelling (< 1%), TEMPO was added as a radical scavenger to stabilize the diaryliodonium salt precursor. Indeed, these types of compounds are known to decompose into radical intermediates upon heating. Although TEMPO is known to be genotoxic, the QC study shows that the presence of traces of this radical in the final injectable solution was far below the specification (500–1000 times). This process, fully automated on an AIO from Trasis, proceeds at the Curie level with an RCY of 35% (dc). The duration of the synthesis is only of 50 min and the molar activity is very high (815 GBq·µmol^−1^). However, even if the affinity of UCB-H is slightly lower compared to UCB-J, leading to more difficulties in detecting the variation of SV2A availability, this radiotracer has a more convenient half live of 110 min and its precursor is commercial available as the dedicated cassette for automated synthesis.

In 2016, [^11^C]UCB-A was labelled with [^11^C]methyl triflate reagent (entry 4) reacting with the triphenylmethyl UCB-A precursor [23]. After an acidic treatment and about 40 min of synthesis, the formulated radiotracer was obtained with a RCY of 14% (dc) and a molar activity of 62.9 GBq.µmol^−1^. Unfortunately, [^11^C]UCB-A showed slow kinetic and slow metabolism with a brain Tmax of 20 min with a maximum of 5 min for the two other radiotracers leading to quantification issue.

The same year, [^11^C]UCB-J was produced by labelling the trifluoroborate precursor with [^11^C]methyl iodide (entry 5) under Suzuki-Miyaura coupling conditions with a palladium catalyst [24]. In a first step, [^11^C]methyl iodide was reacted with the palladium catalyst followed by in situ hydrolysis of the trifluorobrate precursor to activate it. This coupling reaction lead to a 35% decay corrected radiochemical yield and good molar activity (215 GBq·0µmol^−1^). The authors noticed that the efficiency of the labelling by methylation depended from the purity of the precursor. The presence of traces amounts (a few percent) of the corresponding boronic acid was required to obtain good yield. Recently, Rokka et al. changed the solvent of the reaction (THF-H_2_O instead of DMF-H_2_O) and performed the reaction in a single step by mixing all the reagents before the addition of [^11^C] methyl iodide [25]. The RCY of the coupling reaction between [^11^C]methyl iodide and the precursor was 39 ± 5% decay corrected within 40 min with a molar activity of 390 GBq·µmol^−1^ at the end of synthesis. More recently, Sephton et al. proposed a completely automated radiosynthesis of [^11^C]UCB-J with cGMP compliant conditions [26]. The precursor was preactivated with HCl to form the more reactive boronic acid derivative. The RCY of the coupling was 35 ± 4% dc and 11 ± 1 ndc with a molar activity around 30 GBq·µmol^−1^. UCB-J presents the best in class SV2A radiotracer with fast kinetic, high brain uptake and rapid metabolization [24]. However, the low half-life of the carbone-11 (20.4 min) requires on-site cyclotron production resulting in some issue for commercial distribution to PET centers.

To circumvent this half-life issue, Li et al. from Yale University have investigated the feasibility of ^18^F-labelling one of the three fluorine atoms presents on the aromatic ring of the UCB-J molecule [27]. First, the authors tried the traditional ^18^F-fluorine nucleophilic substitution of a halogen leaving group and the isotopic exchange but none of these methods were successful. They managed to obtain [^18^F]UCB-J by ^18^F-labelling an iodonium salt and a iodonium ylide (entry 6). Both reactions generated a racemization of the compound due to the high temperature of the labelling reaction (above 170 °C) requiring a chiral HPLC purification. Those syntheses provided very low RCY (1–2%) and the molar activity was moderate (59 ± 36 GBq·µmol^−1^). Other radiofluorination methods with arylstannanes, phenofluor derivatives and boronic ester were tested without further success. Although [^18^F]UCB-J presented similar imaging properties (clearance, metabolism, kinetic) than [^11^C]UCB-J due to equivalent chemical structure, the radiosynthesis issue led to a need for new SV2A tracers.

In 2018, and a few months apart, the Yale University group and Invicro published a new UCB-J analogue with two fluorine atoms instead of three on the aromatic cycle [28,29]. This compound was initially named [^18^F]SDM-8 by the Yale University group and [^18^F]MNI-1126 by Invicro. The two groups agreed to call it [^18^F]SynVesT-1 (entry 7) for later publication and this name will be used in this paper. This tracer has an affinity comparable to UCB-J (8.4 vs 8.2 respectively) and offers the advantage of a fluorine-18 labelling. Different synthesis route for the preparation of desired tracer were investigated by Li et al. They tried iodonium ylide, boronic ester and arylstannane precursors. The ^18^F-fluorination of the iodonium precursor afforded very low yield (< 1%) partially due to the chiral HPLC purification of the racemate resulting from the high temperature use for the labelling. Note that the authors did not tested the addition of a radical scavenger as TEMPO in the previous study [21]. The boronic ester derivative provided better yields (11% at 150 °C and 6% at 110 °C) but the stannic precursor is the most promising with a RCY of 19% (ndc) after 95min of radiosynthesis and a significant molar activity of 242 MBq·µmol^−1^. The radiolabelling occurred at 110 °C which is lower than the critical racemization temperature of 120 °C. Only the synthesis of the R-enantiomer is reported in this publication. Constantinescu et al. (Invicro) worked also on the tin precursor but they synthesized all the enantiomeric forms of the compound (SynVesT-1 (R), MNI-1128 (S) and the racemate) and compared their PET imaging properties. They managed to obtain RCY between 10 and 20% decay corrected with high specific activities (100–370 GBq·µmol^−1^).

The synthesis of another analogue of UCB-J was also published by the Yale University group. This compound ([^18^F]SMD-2) with only one fluorine atom on the aromatic ring was renamed later in [^18^F]SynVesT-2 [30,31]. Like SynVest-1, SynVesT-2 is also an interesting candidate for SV2A imaging probe. It has been synthesized via an iodonium ylide and an arylstannane precursor. The iodonium approach did not provide high radiochemical yield (1%) but radiosynthesis from the tin precursor provided higher RCY (7%, dc). An average molar activity of 141 Gb·µmol^−1^ was obtained after a 90 min synthesis including the purification and formation processes.

In 2019, a screening of a large panel of potential radioligands for SV2A imaging, was performed to identify compounds with similar binding affinities to UCB-J. From these in vitro homogenate binding, autoradiography and in vivo micro-PET studies [32], it appears that SynVesT-1 and SynVesT-2 are promising candidates for SV2A PET imaging, confirming the previous studies (19–22).

In 2019, the synthesis of a ^18^F-difluoromethyl analogue of UCB-J was reported by Trump et al. [33]. As the substitution of the methyl group presents on the pyridine ring of UCB-J by a fluorine atom slightly reduced the affinity of the molecule for SV2A, these authors investigated the possibility to have a CHF^18^F function instead of the methyl. The radiosynthesis of this difluoromethylated compound [^18^F]**1** (entry 9) implied a late-stage ^18^F-difluoromethylation step. This method proceeds by C-H activation and does not require the synthesis of a specific precursor. The catalyzed addition of the CHF^18^F moiety is generated by UV from a previously synthesized [^18^F]difluorobenzothiazole sulfone. The reaction is not regiospecific and several isomers can be obtained. Even if the RCY (dc) was of only 1.5%, the fully automated process affords sufficient amount of the labelled molecule, with relatively high molar activity (40–80 GBg·µmol^−1^) for subsequent preliminary µPET animal studies [34]. This ^18^F-fluoromethyl labelling approach of *N*-heteroaromatic compounds by C-H activation represents an interesting way to rapidly evaluate new SV2A PET tracers. However, for future PET clinical imaging applications, the radiochemical synthesis of the [^18^F]difluorobenzothiazole sulfone should be improved.

[^11^C]UCB-J and [^18^F]UCB-H are currently the two most widely used radio tracers for SV2A imaging. [^11^C]UCB-J has a higher affinity for the target but the half-life of ^11^C drastically limits its use in PET centers without cyclotron. Conversely, the synthesis of [^18^F]UCB-H is commercially available with good yield and high specific activity. However, due to the limited imaging properties of [^18^F]UCB-H, a new generation of radiotracer has recently been released. [^18^F]SynVesT-1 and [^18^F]SynVesT-2 were produced via labelling of an arylstannane precursor. The disadvantage of this chemistry is the toxicity of organotin compounds as well as copper used as a catalyst which could lead to complications for GMP applications. Indeed, an ICP-MS analysis seems essential to quality control for injection to the patient. On the other hand, the iodonium precursors radiolabelling did not show as interesting yields as for the [^18^F]UCB-H. The use of radical scavenger has not yet been considered, which has however proved its usefulness for the labelling of [^18^F]UCB-H.

## 3. Preclinical Developments of SV2A PET Radiotracers

### 3.1. Drug Metabolism and Pharmacokinetic (DMPK)

The first generation of SV2A PET radiotracers, namely UCB-A, UCB-H and UCB-J shared the same chemical backbone and were profiled with DMPK studies in male Wistar rats (single intravenous injection at 0.1 mg·kg^−1^ for each compound) [13]. We report here the main DMPK features for these three radiotracers.

Among the three compounds, UCB-A displayed good affinity for SV2A protein (Table 1, entry 4) and a good logD value of 1.4 (measured from octanol/water partition coefficient at 25 °C and pH 7.4). The A > B apparent permeability (*P*_app_) was measured on CACO-2 cells and the Efflux ratio (ER) was defined as the ratio of apparent permeabilities (B > A / A > B) with values of 383 nm·s^−1^ and 1.2 respectively. The intrinsic clearance (Clint) measured in human microsomes was 20 µL·min^−1^·mg·protein^−1^. Finally, the fraction unbound in rat brain tissue (Fu% brain) is 12% with a ratio of free brain concentration versus free plasma concentration at 20 min post-administration (Free B/P ratio) is 0.6.

As a comparison, UCB-H and UCB-J displayed a good affinity as well (Table 1, entry 2,3,5). The reported logD values were 2.3 for UCB-H and 2.5 for UCB-J. The *P*_app_/ER were 707 nm·s^−1^ /0.7 for UCB-H and 323 nm·s^−1^ /0.8 for UCB-J. The Clint were 12 µL·min^−1^·mg·protein^−1^ for UCB-H and 16 µL·min^−1^·mg·protein^−1^ for UCB-J. Finally, the Fu% brain and the Free B/P ratio were 8 and 1.6, and 4.5 and 1.6 for UCB-H and UCB-J respectively.

Overall, these data (summarized in Table 2) highlighted that the UCB compounds family have a high permeability and suitable PKPD profile as PET radiotracers. The LogD, a key feature for brain PET radiotracers, is nearly equal for UCB-H and UCB-J, and even lower for UCB-A. In all cases, it is in the suitable range for blood brain barrier crossing. However, another important feature, the free B/P ratio clearly highlights that UCB-H and UCB-J possess the greatest potential (free B/P ratio higher than 1 for both, whereas this ratio is less than 1 for UCB-A).

Concerning the pharmacological profile, UCB-J exhibited a greater than 10-fold and greater than 100-fold selectivity for SV2A over SV2C and SV2B protein, respectively [24]. Regarding UCB-H, the in vivo selectivity for SV2A over the two other isoforms (B and C) was assessed by microPET imaging with pharmacological blocking experiment [35]. The authors performed pharmacological competitions with either vehicle, SV2A competitor (levetiracetam at 10 mg·kg^−1^), SV2B competitor (UCB5203 at 3 mg·kg^−1^), and SV2C competitor (UCB0949 at 3 mg·kg^−1^). Statistical analysis revealed differences between levetiracetam pre-treated group and all the other groups of the study, thereby highlighting the in vivo selectivity of UCB-H for SV2A.

To summarized, all of the three radiotracers initially developed by UCB Pharma displayed very good features for central nervous system PET imaging, with excellent brain penetration and the important requirement that none of them is substrate of P-glycoprotein (P-gp) efflux.

As previously mentioned, the second generation of SV2A PET radiotracers includes mainly the two compounds SynVesT-1 and SynVesT-2. SynVesT-1 seems the most promising compound with an IC50 of 3.52 nM and a logP value measured at 2.32. Plasma free fraction of SynVesT-1 was high, at 43 ± 2% [28]. SynVesT-2 hold a high affinity toward SV2A assessed by Ki values of 7.6 and 12 nM for rat and human SV2A, respectively. Finally, SynVesT-2 possesses a Log D of 2.17 ± 0.02 and a plasma free fraction similar of 41% ± 2% [31].

### 3.2. Preclinical PET Imaging with SV2A Radiotracers

The first in vivo description of SV2A PET radiotracer has been achieved in mice with UCB-H for dosimetric analysis purpose [36]. This study revealed by ex vivo tissue distribution that the brain is one of the mice organs most exposed to radioactive doses, along with the urinary bladder wall and the liver, all the three organs receiving a resulting effective dose of 1.88·10^−^^2^ mSv·MBq^−1^. This study concluded that UCB-H tracer met the standard criteria for radiation exposure in clinical studies with an estimated effective dose of 2.8 mSv for an injected dose of 150 MBq and a maximum injectable dose of approximately 325 MBq per participant. Moreover, this study highlighted the high brain penetration of the tracer in mice.

Afterward, preclinical investigations with UCB-H were conducted in rats (Sprague Dawley) considering that their size better suits microPET brain imaging, as well as images quantification with invasive protocols [19]. Warnock et al. proceeded to a full quantification using an arterial input function (AIF). The AIF was measured thanks to an arteriovenous shunt and a beta-probe. To compute the full kinetic model, they used AIF corrected for plasma-to-whole blood ratio and in vivo metabolism. The metabolism was highly reproducible and fitted a bi-exponential curve. This parent fraction curve revealed that 40% of the UCB-H was metabolized 5 min after the injection, while it remains 20% of the parent compound 30 min after injection. The authors showed that the Logan graphic analysis (start time ranged from 7.5 to 15 min.) afforded a highly reproducible distribution volume (*V*_T_) measurement in a test-retest experimental design. Moreover, they validated the pharmacological blocking by increasing doses of levetiracetam, which ranged from 0.1 to 100 mg·kg^−1^ leading respectively to −9.0 ± 4.6% and −43.8 ± 4.7% decreases in the *V*_T_ value for the whole brain.

This in vivo evaluation of UCB-H as a PET tracer for SV2A protein was followed by several studies that aimed at simplifying the binding quantification, AIF in rats being highly invasive and time consuming. The first step for a non-invasive quantification of UCB-H in rat brain used a population-based input function (PBIF) [37]. The authors averaged 8 individuals AIF to compute the PBIF and validate the used of the PBIF on each individual rat. They reported a high correlation of the *V*_T_ measured either by individual AIF or by the PBIF (R^2^ = 0.99 for all fits, the reported *V*_T_ values for both methods being almost equals for each rat). Beyond the quantification part, the authors used HPLC-MS/MS to identify the *N*-oxide as the main plasmatic metabolite of UCB-H (the UCB-H-*N*-oxide represented 90.2% of the formed metabolites). Then, they proceed to the radiolabelling of this UCB-H-*N*-oxide and performed PET imaging with [^18^F]UCB-H-*N*-oxide thereby establishing the complete absence of UCB-H radiometabolite in the rat brain.

The second simplification step for preclinical studies with UCB-H was the validation of a static PET acquisition and standardized uptake value assessment [38]. The authors compared the two parameters: the *V*_T_ obtained from Logan graphical analysis using the previously developed PBIF, and the standardized uptake value (SUV). The authors used pharmacological competition design with 1 and 10 mg·kg^−1^ of levetiracetam in rats to study the impact of the length of the dynamic acquisition (90 versus 60 min) and the correlations between *V*_T_ and SUV over 20 min consecutive timeframes. They reported no bias between the *V*_T_ values obtained for both dynamic acquisition times, and a high correlation between the *V*_T_ derived from 90 min acquisition time and the SUV computed from 20 to 40 min static acquisition. The authors identified the 20–40 min timeframe as the best situation for a static PET acquisition with UCB-H in rat. This method of static acquisition and SUV assessment was further used to investigate the in vivo modification in SV2A protein expression in a preclinical rat model of temporal lobe epilepsy. Although pathophysiological findings are beyond the scope of this review, this study highlights the efficiency of static PET analysis with UCB-H to detect variations in SV2A expression in the brain of an epileptic rat model, as well as physiological modifications related to brain development and maturation [39].

The UCB-J was initially developed in rhesus monkey [24]. As it has been shown with UCB-H, UCB-J displayed a fast metabolism, the parent fraction in the plasma accounted for approximately 40% and about 25% of the radioactivity at 30 and 90 min after injection, respectively. PET images revealed a high brain uptake throughout the grey matter, consistent with the ubiquitous distribution of SV2A. The pre-injection of levetiracetam (10 mg·kg^−1^) substantially blocked [^11^C]UCB-J binding and co-injection of cold UCB-J (150 mg·kg^−1^) drastically decreased specific UCB-J binding. [^11^C]UCB-J displayed a high uptake and rapid kinetics, with an SUV peak of 5–8 in grey matter areas and peak uptake times ranging from 10 to 50 min. Nabulsi et al. tested several kinetic models to quantify UCB-J binding and chose the 1-Tissu compartmental model (1T model) as it produced reliable *V*_T_ estimates with low variability (compared to the other kinetic analyses tested, the multilinear analysis and the 2-tissues compartmental model). Then, using the 1T model, the author reported a significant reduction in regional *V*_T_ due to levetiracetam pre-injection (10 and 30 mg·kg^−1^). The resulting receptor occupancy analysis revealed that 59% and 89% of SV2A in rhesus monkey brain were occupied by 10 and 30 mg·kg^−1^ of levetiracetam respectively. The blocking experiment using 17, 50, and 150 mg·kg^−1^ of UCB-J showed respectively an occupancy of occupancies of 46%, 68%, and 87%. Thanks to these results, the authors were able to compute the *Kd* value for UCB-J estimated 3.4 ± 0.2 nM and the *B*_max_ for several brain regions (highest value in cingulate cortex 350 nmol·L^−1^, and lowest value in the pons and the brain stem 123 and 124 nmol·L^−1^ respectively).

Regarding the dosimetry, they performed a whole-body distribution studies showing that the liver was receiving the largest doses for males (0.0199 mGy·MBq^−1^) whereas the brain received the largest dose for females (0.0181 mGy·MBq^−1^). They estimated the effective dose equivalent value of approximately 4.5 mSv·MBq^−1^ and conclude that the maximum effective dose equivalent from a single 740 MBq (20 mCi) administration of [^11^C]UCB-J is equivalent to 3.4 mSv, thus being fully compliant with regulation for human PET imaging.

The same group published in 2016 the only complete validation of SV2A PET as marker of synaptic density [40]. This well-designed study was realized in vivo with [^11^C]UCB-J PET imaging in an olive baboon (Papio anubis), after which the animal was sacrificed, and the brain was dissected for post-mortem tissue studies. The post-mortem analyses (involving 12 brain regions) consisted in western blotting, SV2A homogenate binding assays and immunohistochemical staining for SV2A and synaptophysin protein as reference marker for synaptic density measures. To established that SV2A can be used as marker of synaptic density, they compared regional densities of SV2A and the “gold standard” synaptic marker synaptophysin using selective antibodies. SV2A and synaptophysin signals were strong and specific in all grey matter regions but absent or weak in the centrum semiovale (CS). There was an excellent linear correlation between SV2A and synaptophysin across all grey matter regions. They were able to establish that SV2A can be used as an alternative to Synaptophysin for accurate synapse quantification. In addition, the authors established a strong correlation between the in vitro regional distribution of SV2A and the [^11^C]UCB-J *V*_T_ measured in vivo by PET. Afterward, to further evaluate the relationship between in vivo [^11^C]UCB-J binding and SV2A density, homogenate binding studies were performed to determine affinity (*Kd*) and regional SV2A densities *B*_max_. The authors reported a clear correlation between the in vitro *B*_max_ values measured in the 12 brain regions and the in vivo *V*_T_ values. As a cross validation, there was an excellent correlation between the *Kd* derived from homogenate binding and the regional SV2A Western blot measurements.

Recently, UCB-J was investigated in mice and more specifically in the amyloid precursor protein and presenilin 1 double-transgenic (APPswe/PS1DE9 [APP/PS1]) mouse model of Alzheimer disease [41]. The authors intended to perform a longitudinal [^11^C]UCB-J PET on these AD mice to measure the treatment effects of saracatinib, an inhibitor of the tyrosine kinase Fyn which is believed to be useful in AD treatment. The experimental design consisted in the testing of two groups: the control group with wild type (WT) mice and the test group with APPswe/PS1DE9 mice. Both groups underwent three [^11^C]UCB-J PET measurements: at baseline, after treatment, and during drug washout (more than 27 d after the end of treatment). The quantification was achieved with several parameters, the first to be used was the BP_ND_, computed by the simplified reference tissue model with the brain stem as reference region. Then the authors compared this BP_ND_ with the SUVratio using the brain stem signal for normalization (SUVR_BS_, static acquisition time of 30 min between 30 and 60 min post-injection). The authors also tested another SUVratio normalized by the whole brain (SUVR_WB_). The authors found that BP_ND_ and SUVR_BS_ demonstrate excellent agreement with correlation coefficient R^2^ = 0.85 for the whole brain region. However, for treatment response assessment, the authors claimed that SUVR_WB_ gave less variability mainly due to the small size of the used regions of interest. Then, Toyonaga et al. stated that [^11^C]UCB-J PET allows to differentiate hippocampal SUVR_WB_ in APP/PS1 mice and in WT mice at baseline and to detect a significant increase in hippocampal SUVR_WB_ after AD treatment with saracatinib.

In 2016, UCB Pharma, in collaboration with Yale University, reported preclinical data on their newly developed antiepileptic drug named Brivaracetam (acting on SV2A, like its predecessor compound levetiracetam). They used PET imaging to prove a faster SV2A occupancy by brivaracetam compared with levetiracetam [42]. Displacement experiments using [^11^C]UCB-J were performed in rhesus monkeys to estimate the time course of tracer exit from the brain after single IV dosing of brivaracetam (5 mg·kg^−1^) or levetiracetam (30 mg·kg^−1^). The estimated displacement half-times were 10 min for brivaracetam and 30 min for levetiracetam. Further, using kinetic modelling, they were able to predict drug entry half-times, which were 3 min for brivaracetam and 23 min for levetiracetam.

The third SV2A radiotracer belonging to the first generation is the UCB-A [23]. Unlike the two other UCB tracers, UCB-A displayed a relatively slow metabolism, with 93% and 42% intact tracer present at 5 and 40 min post-injection. Surprisingly, the [^11^C]UCB-A showed a slow kinetic profile with an accumulation during the first 60 min, followed by a slow wash out from the brain. The time activity curves of the whole brain clearly demonstrated a successful blocking of [^11^C]UCB-A binding by the pre-injection of an acetamide-based ligand of SV2A (named compound 4 in the study, 10 mg·kg^−1^). Further, the reversible binding of [^11^C]UCB-A was demonstrated by the administration of brivaracetam (21 mg·kg^−1^), at 45 min post-injection, which fully and rapidly displaced [^11^C]UCB-A. Afterward, the [^11^C]UCB-A was further developed in pig where PET images were quantified by whole blood time-activity measures and metabolite-corrected arterial input curves for kinetic modelling. The authors used the Akaike criteria to determine that [^11^C]UCB-A kinetics were best described by a 1T model. With this model, they could estimate both *V*_T_ and *k*_1_ parameters. However, the 1T model was unable to properly estimate *V*_T_ in the blocking condition. The authors found that Logan graphical analysis could reliably fit baseline and blocking conditions and therefore decided to use it in the occupancy analysis of their blocking conditions. Regarding the dosimetry in rats, the organ receiving the highest absorbed dose was the liver, at 12.5 and 26.3 µGy·MBq^−1^ in males and females, respectively. Effective doses were 2.9 µSv·MBq^−1^ and 4.6 μSv·MBq^−1^, for males and females, respectively. The authors claimed that given the effective doses observed in rats, an effective dose of no more than 1.8 mSv per typical administration of 400 MBq would be achievable.

Currently, UCB-J has been fluorinated and tested in vivo in non-human primates [27]. As the chemical structure is the same as UCB-J, all features of UCB-J radiotracer remain the same and the authors stated that the tracer possesses the same in vivo behavior has its parent compounds UCB-J.

The second generation of radiotracer is leaded by two tracers: SynVesT-1 and SynVesT-2. Both tracers display a very high affinity for SV2A (reported previously in this review). Both of them were evaluated in non-human primates [28,31]. SynVesT-1 and SynVesT-2 share a very similar metabolism rate in plasma. The first one displayed 42 ± 13% of parent radiotracer remaining at 30 min after injection, which further decreased to 27 ± 5% and 23 ± 5%, respectively, at 60 and 90 min. post-injection. For the second one, the authors reported that 34% ± 0.1% of intact parent radiotracer remains at 30 min post-injection, which further decreased to 24% ± 3% and 22% ± 4%, at 60 and 90 min, respectively. In rhesus monkeys, both tracer with high tracer uptake in gray matter and very low uptake in white matter (the lower value being found in the CS, as in the case of UCB-J PET imaging). SynVesT-1 has been drastically displaced by the administration of levetiracetam (30 mg·kg^−1^) at 90 min post-injection. The binding specificity was emphasized by blocking studies, either by pretreatment with levetiracetam (30 mg·kg^−1^) or UCB-J (0.15 mg·kg^−1^). Both competitors dramatically decreased regional uptake levels across all brain regions and resulted in earlier peak uptake. Regarding SynVesT-2 preblocking study with unlabelled UCB-J (150 μg·kg^−1^) completely reduced the specific binding of the tracer. Although both tracers look very similar, it is worth mentioning that their brain kinetics largely differ. Indeed, SynVesT-1 displayed an SUV peak within 30 min post-injection in all brain regions followed by a moderate rate of clearance over time, whereas SynVesT-2 showed a much faster brain kinetic with a SUV peak reached at 10 min post-injection. On the contrary, both tracers display a very similar uptake pattern with the highest tissue uptake levels found in the frontal cortex and putamen (SUV > 8 for both), and lowest in CS (SUV < 2 for both). Concerning the quantification of PET images, both tracers were analyzed with either the 1T model or the simplified reference tissue model (SRTM) using the CS as reference tissue. The 1T model allows to compute the *V*_T_ parameter which has proven to be more than two times higher for SynVesT-1 than for SynVesT-2 across all brain regions. In addition, the *V*_T_ values for SynVesT-1 are much closer to those obtained with UCB-J in rhesus monkeys. Similarly, with the BP_ND_ obtained with SRTM, values for SynVesT-1 were higher for all brain regions, but this time BP_ND_ values for SynVesT-2 are closer to those obtained with UCB-J.

In a nutshell, SynVesT-1 and SynVesT-2 share lots of common features in terms of metabolism and in vivo behavior, although SynVesT-1 seems the most promising one with close outcomes to UCB-J PET imaging.

## 4. Clinical Studies with SV2A PET Radiotracers

### 4.1. Quantification of SV2A PET Radiotracers Binding

#### 4.1.1. UCB-H in Human Brain

The distribution of UCB-H in the human brain was studied using the full kinetic modelling approach [43,44]. Authors have motivated their orientation to the full kinetic modelling by the ubiquitous distribution of SV2A in the brain, which makes very unlikely the identification of a “reference region” with all its necessary characteristics for modelling the radiotracer distribution [45]. Moreover, the white matter usually used as reference region for several tracers and studies was also discarded because of the presence of white matter lesions in several elderly and AD participants [46]. In order to overcome the problem of patient discomfort during the arterial input function (AIF) sampling, an alternative method using carotid artery image-derived input function was developed for UCB-H quantification [43]. Even with this image-derived input function, blood sampling was not completely discarded, and authors collected venous blood samples in a group-representative number of subjects at six time-points post injection in order to determine the plasmatic parent fraction needed for image-derived input function correction.

The input function was derived from dynamic PET images [43]. Briefly, the method extracts time series of radiotracer activity in the carotid arteries [47]. The identification of voxels belonging to the carotids is based on the computation of the Pearson product-moment correlation coefficient between a “seeding region” and voxels in a mask containing the carotid. As, during the first 2min, radioactivity is mainly localized in the vessels, inducing a large spill-out effect [47], the signal was corrected for this spill-out effect using the geometric transfer matrix approach [48]. The extracted time-series signal was then corrected for the mean unchanged plasma fraction and used for kinetic modelling.

As image analysis and kinetic modelling, UCB-H PET dynamic data frames underwent a series of processing steps before voxel-wise parametric map extraction. Indeed, data frames were corrected for participant’s motion during the acquisition and co-registered to the corresponding MRI structural image (the sum of frames between 2 and 30 min as source image), and then partial volume corrected. The PET scanner low resolutions as well the small structures of interest (e.g., hippocampus and the basal forebrain that are atrophied in early Alzheimer’s disease are the main raisons for partial volume correction [49]. The iterative Yang (PETPVC toolbox, [50]) voxel-wise PVC method was used, with grey matter, white matter, CSF as ROI mask. Kinetic modelling using PVE-corrected dynamic PET data and image-derived input function was done with PMOD software (PMOD Technologies, Zurich, Switzerland). Logan graphical analysis method was used to calculate the *V*_T_ map of UCB-H in the brain with t* of 25 min. The obtained *V*_T_ map was then spatially normalized into the MNI space using the transformation parameters obtained during structural MRI spatial normalization. Spatial normalization is usually done through the structural MRI image as the last one has a higher resolution. Mean regional *V*_T_ values were then extracted from *V*_T_ map using the AAL atlas [51].

#### 4.1.2. UCB-J in Human Brain

UCB-J in vivo quantification in human brain was presented in the publication of Finnema 2016 where the authors proposed the CS as a reference region for non-displaceable binding [40]. Regional non-displaceable binding potential (BP_ND_) was calculated as the ratio between the distribution volume in region of interest and the distribution volume in the CS minus one (*V*_T,region_ / *V*_T,centrum_semiovale_ −1). The volume of distribution was calculated using a full kinetic modelling through the 1T model with measured arterial input function and regional time activity curves (TACs). The extraction of the regional time activity curves was done on the subject PET space using AAL atlas. Briefly, the inverse transformation parameters obtained during the spatial normalization of the motion corrected UCB-J dynamic data were applied to the AAL atlas to bring it into the PET space. Then, regional TACs were extracted end expressed in SUV by normalization of the regional activity concentration by the ratio of injected dose to subject weight.

Parametric BPND maps were also generated with the simplified reference tissue model 2 using the CS as a reference region [52]. The author showed that CS has some degree of specific binding and further follow-up studies and analysis were still needed to justify its use as a reference region.

In a recent study aiming at the validation of the CS, Rossano et al. investigated the region of interest definition and the reconstruction parameters (number of iteration of the ordered subset expectation minimization “OS-EM” [53]. Indeed, optimization of the CS ROI definition aims to minimize the inclusion of grey matter and partial volume effects that would lead to erroneous CS *V*_T_ values. This was done using an improved ROI definition strategy based on the use of the CS AAL to mask the individual white matter and the definition of a group CS ROIs with increasing volume (2, 4, and 6 mL). Additionally, convergence using the OS-EM algorithm was investigated by increasing the iterations used to reconstruct the PET image, which would minimize any positive bias that may be observed in the low-intensity white matter, surrounded by the high-intensity grey matter. Moreover, baseline and blocking (levetiracetam and brivaracetam) UCB-J scans were used to estimate the V_ND_ of the grey matter, and the validity of using CS *V*_T_ to estimate V_ND_ in calculating BP_ND_ was evaluated. Even if the results showed a specific uptake in the white matter lower than 10% of that in the grey matter and the CS uptake was predominantly due to non-displaceable binding, the authors stressed the importance of assessing differences in white matter uptake when investigating differences across disease groups, especially in diseases with white matter pathology. Koole et al. also investigated CS as reference region for UCB-J quantification showing considerable agreement with Rossano conclusions [54]. Compared to a full kinetic analysis, with 1T compartment method with measured AIF, simplified reference tissue model approach using a 90 min acquisition interval and the CS as reference tissue provided a negligible bias of less than 6%. Moreover, the authors suggested shortening the dynamic acquisition time to 70 min instead of 120 min. Alternately to dynamic tracer uptake modelling, SUV ratio relative to the CS for 30 min acquisition starting 60 min after tracer injection was proposed as a quantitative approximation for UCB-J brain PET imaging. The use of CS white matter as reference region for [^11^C]UCB-J quantification has been further supported by in vitro autoradiographic observations of low binding for [^11^C]UCB-J in this region, in either human non-human primates [55].

### 4.2. Clinical Outcomes with SV2A PET Imaging

**Figure 2:**
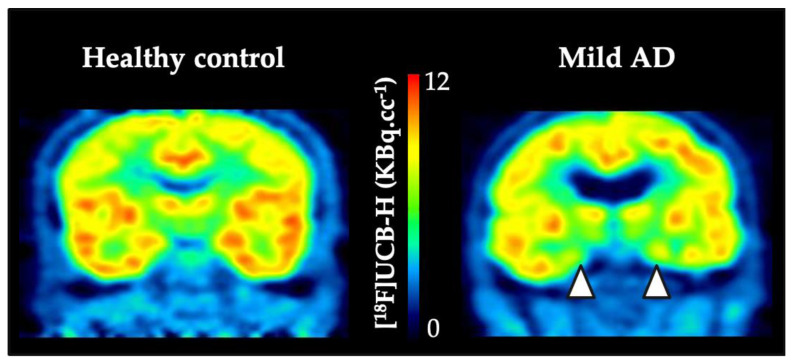
Individual example of [^18^F]UCB-H in healthy control and in mild AD patient. The white arrow heads highlight the reduced [^18^F]UCB-H uptake in temporal lobe areas of the mild AD patient.

The first in-human study with SV2A specific PET tracer was published by Bretin et al. and concerned biodistribution description and radiation dosimetry assessment [56]. The authors reported the results of dynamic whole-body PET with UCB-H on five healthy males with an injected activity between 139.1–156.5 MBq. The radiation dosimetry was calculated using OLINDA/EXM. Regarding the brain, as main organ of interest, the absorbed dose was 1.89×10^−2^ ± 2.32 × 10^−3^ mGy·MBq^−1^. The methodological aspects of UCB-H brain images quantification have been described above in this review [43]. Thanks to the implementation of the image-derived blood input function, the same group reported the outcomes of UCB-H PET imaging in twenty-four patients with mild cognitive impairment or Alzheimer’s disease (with positive [^18^F]Flutemetamol amyloid-PET) compared to 19 healthy controls (Figure 2., [44]). Using voxel-wise analysis on *V*_T_ maps after partial volume effect correction (amyloid positive patients vs control group), the authors described a significant reduction of synaptic density in the right anterior hippocampus extending to the entorhinal cortex (26.9% decrease in right hippocampal [^18^F]UCB-H *V*_T_). A sub-analysis focusing only on mild AD patient (n = 14) showed reduced synaptic density in the right superior temporal gyrus. Finally, the authors computed Pearson correlations between [^18^F]UCB-H values in selected ROIs and cognitive measures. When taking into account the correction for multiple comparisons, the authors showed correlations between awareness of memory problems and MMSE scores on the one hand and [^18^F]UCB-H values in the hippocampus on the other hand (Pearson correlation coefficient of −0.75 and 0.57 respectively, at *p* < 0.005). Thus, Bastin et al. clearly confirmed that SV2A-PET imaging with [^18^F]UCB-H, allows to image in vivo synaptic changes in Aβ-positive patients with neurocognitive disorder and to relate them to cognitive impairment with regional specificity according to the cognitive domain.

The first study reporting SV2A PET assessment in AD was published by the University of Yale and used UCB-J [57]. The outcomes parameters used as imaging outcomes were the *V*_T_, the delivery rate constant K_1_ and the BP_ND_ (obtained with the 1T and the SRTM 2 models). The study included twenty-one participants, with 10 amnestic MCI due to AD or mild AD dementia participants and 11 healthy controls. The results were consistent with the hypothesis derived from AD Braak staging, showing that [^11^C]UCB-J binding was significantly reduced in the hippocampus of participants with AD compared to the control group. In the hippocampus of AD participants, the *V*_T_ was 28% lower and the BP_ND_ was reduced by 44%. Parametric map of K1 value, reflecting the cerebral blood flow clearly highlighted that the pattern of regional *k*_1_ reduction in the participant with AD was similar to that of hypometabolism observed with FDG-PET in AD. Finally, Chen et al. reported Pearson correlations between [^11^C]UCB-J values in the hippocampus and cognitive measures. Notably, statistically significant correlations were found between SRTM2-derived hippocampus BP_ND_ and episodic memory on one hand (R = 0.56; *p* =0.01), and with clinical dementia rating–sum of boxes (CDR-SB) score on the other hand (R = −0.61; *p* =0.003). These results were later confirmed in a study conducted by the group of KU Leuven where prodromal AD patients (aMCI) were tested with both [^11^C]UCB-J and [^18^F]MK-6240 that allows the assessment of Tau protein deposition [58]. Within the median temporal lobe, there was a clear correlation between [^11^C]UCB-J SUVR decrease and [^18^F]MK-6240 SUVR increase (r = −0.076, *p* = 0.02). The authors corroborated previous results with SV2A PET imaging while highlighting the hippocampus as the most prominent region for synapse loss. Moreover, synaptic loss correlates with cognitive decline measured by MMSE score and episodic memory test (r = 0.77 and 0.7 respectively).

As a logical follow-up of the preclinical study that supported the development of the brivaracetam anti-epileptic drug, UCB Pharma and Yale University released a drug occupancy study in human for brivaracetam using [^11^C]UCB-J PET [59]. Three cohorts with four, five, and four healthy subjects were tested for baseline and displacement, displacement and 4 h post-dose and steady state of oral dosing of brivaracetam respectively. The [^11^C]UCB-J PET scanning procedure consisted of an intravenous bolus plus constant infusion. The regional *V*_T_ parameter was derived from 1T model and SV2A occupancy computed by regressing the change in *V*_T_ against the baseline *V*_T_ across regions. The main occupancy results were 66% and 70% for intravenous 100 mg of brivaracetam, 84% and 85% for intravenous 200 mg of brivaracetam, and 78–84% for intravenous 1500 mg of levetiracetam approximately 4 h post-dose. Moreover, the combined IC_50_ estimate across Cohorts 2 and 3 was 0.46 μg·mL^−1^ for brivaracetam and 4.02 μg·mL^−1^ for levetiracetam, leading the authors to state that brivaracetam has 8.7-fold higher affinity for SV2A than levetiracetam.

Recently, the group of Yale University published a PET imaging study with [^11^C]UCB-J in a cohort of Parkinson’s disease patients [60]. Based on current pathophysiological knowledge of PD, more precisely on the fact that synaptic changes are centrally involved in PD and characteristic of the disease pathogenesis, the authors seek to detect synaptic loss in vivo with [^11^C]UCB-J PET in individuals with mild bilateral PD [61]. Twelve PD subjects, assessed according to the Movement Disorders Society Unified PD Rating Scale, were compared to 12 matched normal controls. As previously described, the PET data were processed using SRTM2 kinetic modelling with BP_ND_ as imaging outcome. Statistical lower SV2A-specific binding values were found in PD subjects in several brain regions, the substantia nigra (−45%; *p* <0.001), red nucleus (−31%; *p* =0.03), locus coeruleus (−17%; *p* =0.03), and parahippocampal gyrus (−12%; *p* <0.01). The lower SV2A binding in the substantia nigra was further confirmed by in vitro autoradiography (−17%; *p* <0.005). The authors concluded that their results were in line with the hypothesis claiming that clinical symptomatology of PD may begin in the synapses of brainstem nuclei and remain present throughout the course of the disease [61].

Concerning psychiatric disorders, a recent article reported the in vivo investigation of synaptic density in schizophrenia [62]. This study is in line with evidences implicating synaptic dysfunction in the pathophysiology of schizophrenia. The authors included 18 individuals with a DSM-5 diagnosis of schizophrenia and compared them to 18 healthy volunteers using [^11^C]UCB-J *V*_T_, derived from the 1T model, as imaging outcome. Post-hoc analyses revealed that mean [^11^C]UCB-J *V*_T_ (ml·cm^−3^) was significantly reduced in schizophrenic patients relative to the control group in the frontal cortex (t = 2.51, df = 34.0, *p* =0.03), and in the anterior cingulate cortex (t = 2.83, df = 34.0, *p* =0.02) with large effect sizes (Cohen’s d = 0.8 and 0.9 respectively). Interestingly, Onwordi et al. computed the distribution volume ratio (DVR, using the CS as reference region) in the selected ROIs and found a significant reduction in the hippocampus, in addition to the frontal cortex (FC) and the anterior cingulate cortex (ACC). This discrepancy was analyzed in the sense that DVR values show lower variability and so are likely to have greater sensitivity to detect group differences. Furthermore, the authors stated the absence of significant associations between [^11^C]UCB-J *V*_T_ and corrected grey matter volume in any of the analyzed ROIs, as well as between [^11^C]UCB-J *V*_T_ in the FC, ACC and the assessed clinical variables. The authors completed their clinical results by preclinical in vitro experiment that showed the absence of effect of chronic haloperidol administration on SV2A levels in the rat frontal cortex. This study raised the question whether these results rely on specific loss of SV2A protein or indicate lower synaptic terminal levels. So far, there is no evidence that antipsychotic drugs act on synaptic density, but it would be clinically relevant to compare longitudinally patients who do not respond to antipsychotic treatments and patients who do so.

In parallel, the group from Yale university investigated major depressive disorder (MDD) and post-traumatic stress disorder (PTSD) with the hypothesis that synaptic density, measured by [^11^C]UCB-J PET, would be negatively associated with severity of depressive symptoms [63]. The study included twenty-six unmedicated clinical subjects and twenty-one age, sex, and smoking-matched healthy control subjects. The [^11^C]UCB-J *V*_T_ derived from 1T model after partial volume correction used to correct for effects of tissue atrophy. Three regions of interest were considered: the hippocampus, the dorsolateral prefrontal cortex (dlPFC), and the anterior cingulate cortex (ACC). In each of these regions, a significant lower [^11^C]UCB-J *V*_T_ in individuals with high severity depressive symptoms was detected in comparison to healthy controls. Strikingly, the authors reported significant negative correlations between the [^11^C]UCB-J *V*_T_ and the severity of depressive symptoms (scored with the HAMD-17 scale) across all clinical subjects in dlPFC (r = −0.633, *p* = 0.001), ACC (r = −0.634, *p* = 0.001) and hippocampus (r = −0.487, *p* = 0.012). Finally, Holmes et al. looked at the functional connectivity in the study cohort and performed voxel-wise correlations to associate the seed connectivity data from the dlPFC to synaptic density. They found a significant negative correlation between dlPFC-posterior cingulate cortex (PCC) connectivity and SV2A density in the dlPFC in the clinical group (r = −0.60, *p* = 0.002). The authors concluded that their results prompt the discovery and evaluation of new treatments that should target synaptic density to increase synaptic connections.

Overall, the clinical outcomes of SV2A PET imaging suggest that this technic might bring new outcomes in clinical trials for disease monitoring and treatment response assessment, as well as in the refinement of the studies by providing new inclusion/exclusion criteria.

## 5. Conclusions

Currently, [^11^C]UCB-J and [^18^F]UCB-H are the two most widely used radiotracers for SV2A PET imaging in patients with neurological or psychiatric disorders, but they both present disadvantages. On one hand, the half-life of ^11^C drastically limits the availability of [^11^C]UCB-J, and [^18^F]UCB-H provides a lower specific binding signal on the other hand. Notwithstanding, clinical outcomes obtained with both of them were very coherent with each other. The second generation of SV2A tracers is leaded by [^18^F]SynVesT-1 and [^18^F]SynVesT-2 which are both produced via labelling of an arylstannane precursor. The use of organotin compounds required ICP-MS analysis to ensure the injectability to the patients. Improvements of the radiochemical yield of iodonium precursors labelling could be an alternative to this stannic derivatives limitation. Alternatively, another radiotracer, a ^18^F-difluoromethyl analogue of UCB-J that can be produced by C-H activation, does not require the synthesis of a specific precursor. However, for future PET clinical imaging applications, the radiochemical synthesis should also be improved. Nevertheless, and despite the quality of the newly designed fluorinated radiotracers, SV2A PET imaging still faces its biggest challenge, that is, in being included in a clinical routine for patient care and management.

## Figures and Tables

**Table 1:** Affinity, molar activity and radiochemical yield (RCY) of published SV2A tracers.

Entry	Tracer	Ref	Synthesis of the Radiotracer	pIC_50_ for Human SV2A	Ki (nM) for Human SV2A	Molar Activity (GBq. µmol^−1^)	RCY (%)
1	[^11^C]Levetir-acetam	[18]	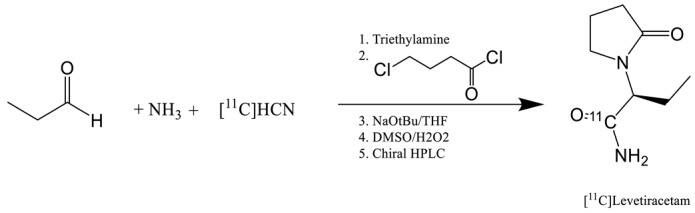	5.7 ^[24]^	2500	17	8.3 (dc)
2	[^18^F]UCB-H	[19]	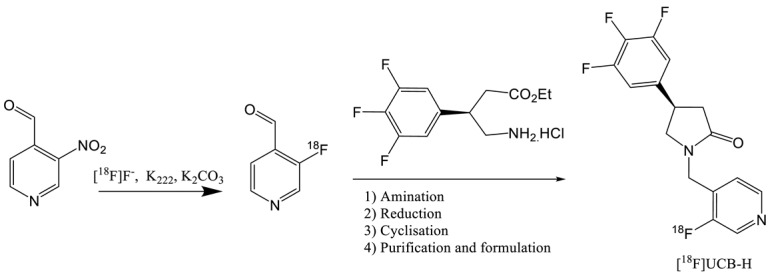	7.8	9.0	518	15 (ndc)
3		[21]	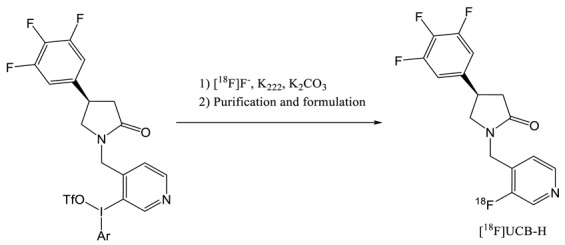	815 ± 185	35 (ndc)
4	[^18^F]UCB-A	[23]	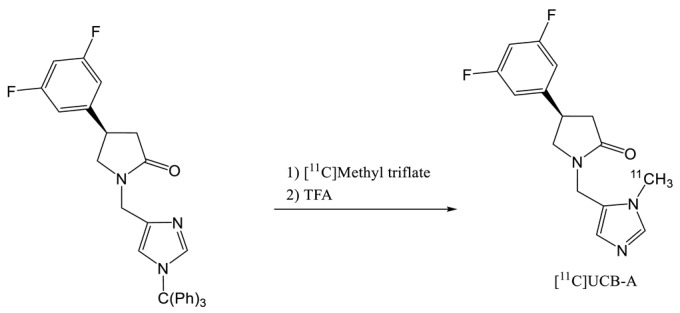	7.9 ^[24]^	ND ^a^	65	14 (dc)
5	[^11^C]UCB-J	[24]	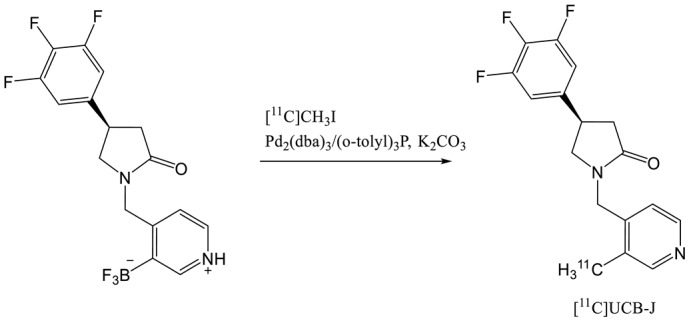	8.2	1.5	215	35 (dc)
6	[^18^F]UCB-J	[27]	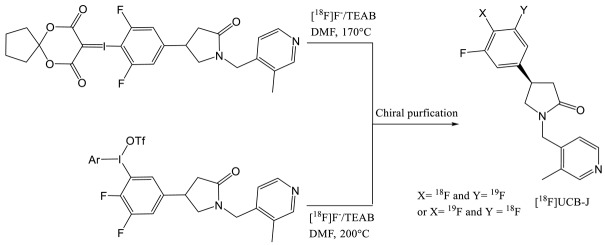	Similar to [^11^C]UCB-J	Similar to [^11^C]UCB-J	59 ± 36	1–2 (ndc)
7	[^18^F]SynVesT-1	[28,29]	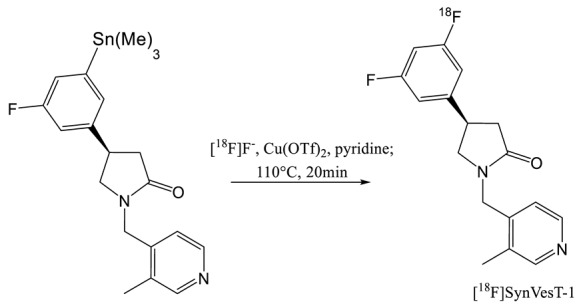	8.4	2.2–4.7 ^b^	242	19 (ndc)
8	[^18^F]SynVesT-2	[31]	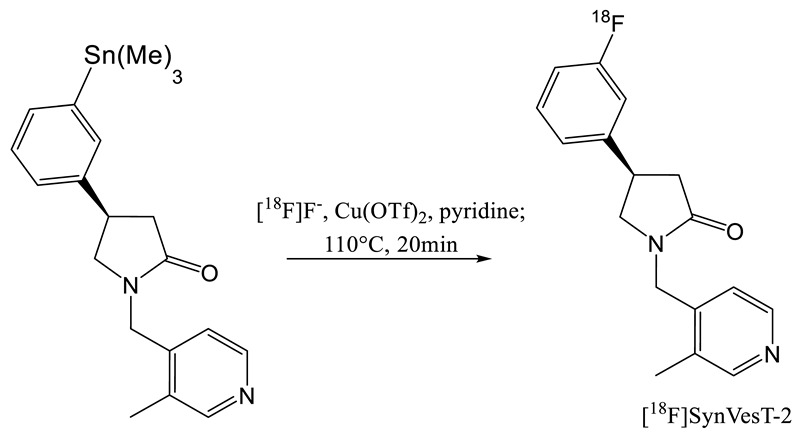	ND ^a^	12	141	7(dc)
9	[^18^F]1	[33]	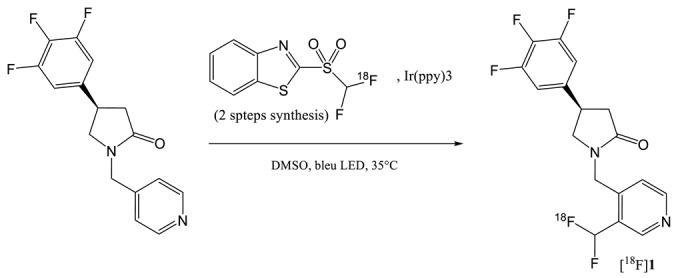	8.3	ND ^a^	40–80	1.5 (dc)

^a^ Unpublished information. ^b^ Respectively the value obtained by Invicro and Yale University.

**Table 2:** Main PK/PD characteristics of the first-generation of SV2A radiotracers derived from IUCB Pharma library. LogP measured from octanol/water partition coefficient at 258C and pH 7.4, Efflux ratio (ER) derived from apparent permeabilities, the intrinsic clearance (Cl*int*) measured in human microsomes, the fraction unbound in rat brain tissue (Fu% brain) and the ratio of free brain concentration versus free plasma concentration (Free B/P ratio).

Tracers	LogD	ER	Cl*int* µL·min^−1^·mg·Protein^−1^	Fu% Brain	Free B/P Ratio
[^11^C]UCB-A	1.4	1.2	20	12	0.6
[^18^F]UCB-H	2.3	0.7	12	8	1.6
[^11^C]UCB-J	2.5	0.8	16	4.5	1.6
